# Are *MTHFR* C677T and *MTRR* A66G Polymorphisms Associated with Overweight/Obesity Risk? From a Case-Control to a Meta-Analysis of 30,327 Subjects

**DOI:** 10.3390/ijms160611849

**Published:** 2015-05-26

**Authors:** Shu-Jun Fan, Bo-Yi Yang, Xue-Yuan Zhi, Miao He, Da Wang, Yan-Xun Wang, Yi-Nuo Wang, Jian Wei, Quan-Mei Zheng, Gui-Fan Sun

**Affiliations:** 1 Research Center of Environment and Non-Communicable Disease, School of Public Health, China Medical University, Shenyang 110013, China; E-Mails: fanfan0721ykl@163.com (S.-J.F.); boyiyangcmu@163.com (B.-Y.Y.); zhixy90smile@126.com (X.-Y.Z.); hemiao.cmu@gmail.com (M.H.); 472594283@163.com (D.W.); qmzheng@mail.cmu.edu.cn (Q.-M.Z.); 2 Division of Molecular Preventive Medicine, Shanghai Institute of Targeted Therapy and Molecular Medicine, Shanghai 200433, China; E-Mails: 13817895706@163.com (Y.-X.W.); wangyanxun@genechina.com (Y.-N.W.); 3 Brain Disease Center, Tianjin Dagang Oil Field General Hospital, Tianjin 300280, China; E-Mail: weijiantianjin@163.com (J.W.)

**Keywords:** *MTHFR*, *MTRR*, overweight, obesity, polymorphism

## Abstract

Several studies have examined the associations of methylenetetrahydrofolate reductase (*MTHFR*) C677T and methionine synthase reductase (*MTRR*) A66G polymorphisms with being overweight/obesity. However, the results are still controversial. We therefore conducted a case-control study (517 cases and 741 controls) in a Chinese Han population and then performed a meta-analysis by combining previous studies (5431 cases and 24,896 controls). In our case-control study, the *MTHFR* C677T polymorphism was not significantly associated with being overweight/obesity when examining homozygous codominant, heterozygous codominant, dominant, recessive and allelic genetic models. The following meta-analysis confirmed our case-control results. Heterogeneity was minimal in the overall analysis, and sensitivity analyses and publication bias tests indicated that the meta-analytic results were reliable. Similarly, both the case-control study and meta-analysis found no significant association between the *MTRR* A66G polymorphism and being overweight/obesity. However, sensitivity analyses showed that the associations between the *MTRR* A66G polymorphism and being overweight/obesity became significant in the dominant, heterozygous codominant and allelic models after excluding our case-control study. The results from our case-control study and meta-analysis suggest that both of the two polymorphisms are not associated with being overweight/obesity. Further large-scale population-based studies, especially for the *MTRR* A66G polymorphism, are still needed to confirm or refute our findings.

## 1. Introduction

Being overweight/obesity has become a serious public health problem worldwide, which could increase the likelihood of many diseases, such as diabetes mellitus, cardiovascular diseases and some cancers [1]. According to the International Obesity Taskforce, in 2010, approximately one billion adults globally were overweight, and a further 475 million were obese [2]. The development of being overweight/obesity is interactively influenced by numerous factors, and 40%–70% of the variation of body mass index (BMI) could be attributed to genetic determinants. Therefore, identification of genetic factors causing overweight/obesity may be useful not only in understanding the pathogenesis of the disorder, but also in providing more effective therapeutic and preventive strategies.

In the past few decades, many genes and polymorphisms have been hypothesized to be involved in the pathogenesis of being overweight/obesity [3]. Among them, the C677T polymorphism in the methylenetetrahydrofolate reductase (*MTHFR*) gene and the A66G polymorphism in the methionine synthase reductase (*MTRR*) gene were assessed as potential candidates [4,5,6,7,8,9,10,11,12]. The *MTHFR* irreversibly catalyzes the conversion of 5,10-methylenetetrahydrofolate to 5-methyltetrahydrofolate, which serves as a methyl donor in the remethylation of homocysteine to methionine [13]. The *MTRR* is responsible for the remethylation of homocysteine to methionine via a vitamin B_12_-dependent reaction [14]. Both the *MTHFR* C677T and *MTRR* A66G polymorphisms can affect the activities of their corresponding enzymes and ultimately lead to elevated homocysteine levels [15,16,17]. Several epidemiological studies have reported higher homocysteine or lower folate levels in overweight/obesity subjects compared with normal weight controls [18,19,20]. The mechanisms underlying these observations remain unclear; however, some investigators have postulated that elevated homocysteine levels might affect the development of being overweight/obesity via epigenetic control of gene expression in the regulation of body fat storage, because the methyl group and homocysteine metabolism are interrelated processes and are closely related to methylation of DNA and of amino acid residues on histones [5,6,21,22]. Recent data from animal experiments, cell studies and genetic studies seem to support this hypothesis [23,24,25].

Prior studies have explored the relationships of the *MTHFR* C677T and *MTRR* A66G polymorphisms with being overweight/obesity, but with conflicting results in different ethnic populations [4,5,6,7,8,9,10,11,12]. No significant association of the *MTHFR* C677T polymorphism with being overweight/obesity was observed in Danish [6], Tunisian [9], Iranian [8], Saudi [7], Italian [5] and Thai [4] populations. A genome-wide association study of obesity-related traits also did not observe significant associations of nine polymorphisms in the *MTHFR* gene with BMI, weight or hip circumference [26]. However, two studies carried out, respectively, in England and India, revealed that the 677T allele or 677TT genotype was involved in significantly increased risk of obesity [6,12]. The *MTRR* A66G polymorphism was not as well studied as the *MTHFR* C677T polymorphism. We systematically searched seven databases and found that only three studies have evaluated the relationship of the *MTRR* A66G polymorphism with obesity to date. The *MTRR* 66G allele was found to be significantly associated with an increased risk of obesity among Italians [5] and Indians [12], but the direction of the association was reverse in a Hungarian population [27]. In China, due to changing lifestyles and Westernized dietary habits, the prevalence of being overweight/obesity and its related non-communicable diseases have increased dramatically in recent years. Additionally, our group previously found that the prevalence of the *MTHFR* 677T allele and 677TT genotype was very high in the Chinese population, especially in northerners [28]. Thus, the exploration of the relationships between these polymorphisms and being overweight/obesity among the Chinese population is of significance. However, as far as we know, no such study has been performed in the Chinese population. Therefore, we designed a case-control study to evaluate the associations of these polymorphisms with being overweight/obesity in a northern Chinese Han population. Subsequently, we conducted a comprehensive meta-analysis combining the present study and previously published studies to provide more convincing evidence for the associations between the *MTHFR* C677T and *MTRR* A66G polymorphisms and overweight/obesity risk.

## 2. Results

### 2.1. Population Characteristics in Our Case-Control Study

The demographic and clinical characteristics of the study subjects are summarized in Table 1. Because the average age of the overweight/obesity subjects was greater than that of normal subjects and the sex was not matched between the two groups, clinical data were analyzed using analysis of covariance with age and sex as covariates. As expected, the overweight/obesity subjects manifested significantly higher BMI, systolic blood pressure (SBP), diastolic blood pressure (DBP), waist circumference (WC), fasting blood glucose (FBG), total cholesterol (TC), triglycerides (TG) and low density lipoprotein cholesterol (LDL-C) levels, but lower high density lipoprotein cholesterol (HDL-C) levels as compared with that of controls.

### 2.2. Genotype Distribution and Association Analysis in Our Case-Control Study

Genotypic and allelic frequencies of the *MTHFR* C677T and *MTRR* A66G polymorphisms are shown in Table 2. For the *MTHFR* C677T polymorphism, the genotype distribution in the control group was consistent with Hardy–Weinberg equilibrium (HWE) (*p* = 0.662). The 677T allele and 677TT genotype frequencies were not significantly different between cases and controls (*p =* 0.602 and 0.454, respectively). Under the multivariate logistic regression model adjusted for age and sex, no significant association was found between the *MTHFR* C677T polymorphism and being overweight/obesity when examining homozygous codominant (odds ratio (OR) = 1.06, 95% confidence interval (CI) = 0.75–1.51, *p* = 0.738), heterozygous codominant (OR = 0.83, 95% CI = 0.59–1.08, *p* = 0.308), dominant (OR = 0.92, 95% CI = 0.65–1.32, *p* = 0.655), recessive (OR = 1.21, 95% CI = 0.88–1.67, *p* = 0.245) and allelic (OR = 1.05, 95% CI = 0.85–1.30, *p* = 0.626) models (Table 2). For the *MTRR* A66G polymorphism, the genotype distribution in the control group did not comply with HWE (*p* = 0.045), which might be attributable to low 66GG genotype frequency and, selection bias in controls owing to a hospital-based case-control study, and there might be a selective pressure acting upon this gene locus. The frequencies of the 66G allele and 66GG genotype were not significantly different between cases and controls (*p* = 0.871 and 0.689, respectively). Logistic regression analysis also showed no significant relationship between the polymorphism and being overweight/obesity in homozygous codominant (OR = 0.82, 95% CI = 0.53–1.25, *p* = 0.344), heterozygous codominant (OR = 1.15, 95% CI = 0.92–1.43, *p* = 0.216), dominant (OR = 1.17, 95% CI = 0.87–1.58, *p* = 0.291), recessive (OR = 1.00, 95% CI = 0.56–1.76, *p* = 0.968) and allelic (OR = 1.11, 95% CI = 0.87–1.41, *p* = 0.403) models (Table 2).

**Table 1:** Anthropometric and clinical characteristics of the study subjects.

Characteristics	Controls	Overweight/Obesity	*p*-Value
Number of subjects	741	517	-
Gender (male/female)	369/372	261/256	0.819
Age (year)	44.57 ± 9.71	47.4 ± 9.72	<0.001
Height (cm)	166.81 ± 7.41	166.74 ± 8.23	0.877
Weight (kg)	61.14 ± 6.94	75.31 ± 10.34	<0.001
BMI (kg/m^2^)	21.92 ± 1.39	27.02 ± 2.56	<0.001
WC (cm)	78.16 ± 6.67	90.22 ± 8.32	<0.001
SBP (mmHg)	122.36 ± 17.17	133.89 ± 19.08	<0.001
DBP (mmHg)	77.25 ± 10.77	84.33 ± 12.63	<0.001
FBG (mmol/L)	5.03 ± 0.70	5.27 ± 0.88	<0.001
TC (mmol/L)	4.85 ± 1.02	5.05 ± 0.99	0.001
TG (mmol/L)	1.01 ± 1.00	1.56 ± 0.98	<0.001
HDL-C (mmol/L)	1.31 ± 0.35	1.19 ± 0.30	<0.001
LDL-C (mmol/L)	2.67 ± 0.93	2.80 ± 0.94	0.016

Abbreviations: BMI, body mass index; WC, waist circumference; SBP, systolic blood pressure; DBP, diastolic blood pressure; FBG, fasting blood glucose; TC, cholesterol; TG, triglycerides; HDL-C, high-density lipoprotein cholesterol; LDL-C, low-density lipoprotein cholesterol.

**Table 2:** Association of the *MTHFR* C677T and *MTRR* A66G polymorphisms with overweight/obesity risk.

Polymorphism	Cases (*n* = 517)	Controls (*n* = 741)	Crude OR (95% CI)	*p*-Value	Adjusted OR ^a^	*p*-Value
*MTHFR* C677T						
CC	115 (22.2)	160 (21.6)	1.00	-	1.00	-
CT	244 (47.2)	375 (50.6)	0.91 (0.68–1.21)	0.499	0.83 (0.59–1.18)	0.308
TT	158 (30.6)	206 (27.8)	1.07 (0.78–1.47)	0.688	1.06 (0.75–1.51)	0.738
Allelic model	-	-	1.04 (0.89–1.22)	0.602	1.05 (0.85–1.30)	0.626
Dominant model	-	-	0.96 (0.73–1.26)	0.783	0.92 (0.65–1.32)	0.655
Recessive model	-	-	1.14 (0.89–1.46)	0.288	1.21 (0.88–1.67)	0.245
*MTRR* A66G						
AA	298 (57.6)	437 (59.0)	1.00	-	1.00	-
AG	186 (36.0)	251 (33.9)	1.09 (0.86–1.38)	0.497	1.15 (0.92–1.43)	0.216
GG	33 (6.4)	53 (7.1)	0.91 (0.58–1.45)	0.698	0.82 (0.53–1.25)	0.344
Allelic model	-	-	1.02 (0.84–1.22)	0.871	1.11 (0.87–1.41)	0.403
Dominant model	-	-	1.06 (0.84–1.33)	0.637	1.17 (0.87–1.58)	0.291
Recessive model	-	-	0.89 (0.56–1.39)	0.595	0.99 (0.56–1.76)	0.968

Abbreviations: *MTHFR*, methylenetetrahydrofolate reductase; *MTRR*, methionine synthase reductase; OR, odds ratio; ^a^ adjusted by sex and age.

### 2.3. Meta-Analysis Results

**Figure 1:**
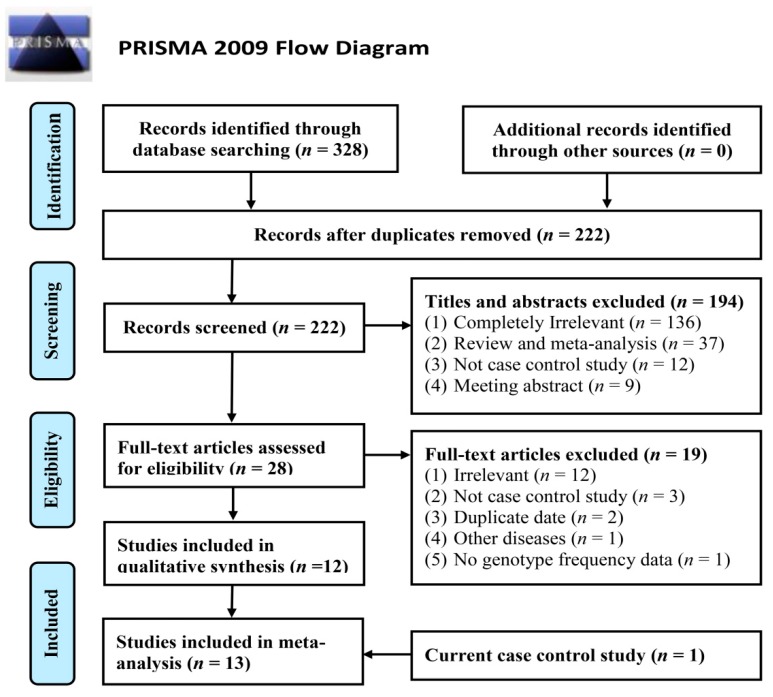
Flow diagram of the study selection process in this meta-analysis.

Figure 1 details the process of selecting and excluding articles. A total of nine publications [4,5,6,7,8,9,10,11,12] with 13 studies (combining the current study) comprising 5431 cases and 24,896 controls were included in the meta-analysis. Characteristics and genotype distributions of these studies are summarized in Table S1 and Table 3, respectively.

Thirteen studies were included in the meta-analysis of the *MTHFR* C677T polymorphism. The pooled results indicated that the *MTHFR* C677T polymorphism was not significantly associated with the risk of being overweight/obesity in all genetic models (recessive model: OR = 1.05, 95% CI = 0.94–1.16, *p* = 0.411; dominant model: OR = 1.06, 95% CI = 1.00–1.13, *p* = 0.092; homozygous codominant model: OR = 1.06, 95% CI = 0.94–1.18, *p* = 0.341; heterozygous codominant model: OR = 1.06, 95% CI = 0.99–1.13, *p* = 0.588; allelic model: OR = 1.05, 95% CI = 1.00–1.10, *p* = 0.077) (Table 4, Figures S1–S5). Between-study heterogeneity was minimal under all genetic models. Stratified analyses based on ethnicity, source of controls, genotyping method and sample size also showed no significant relationship (Table 4). Sensitivity analyses showed that pooled ORs were not substantially influenced by removing each individual study or some specific studies. The shapes of funnel plots in all of the genetic models appeared to be symmetrical (Figure S6), and statistical results did not show any evidence of publication bias (all *p* > 0.05).

**Table 3:** Genotypic and allelic distributions of *MTHFR* C677T and *MTRR* A66G polymorphisms used in the meta-analysis.

Study	Genotype Distribution						Allele Frequency				HWE Test		Number of Cases/Controls
First Author (Year)	Cases			Controls			Cases		Controls		χ^2^	*p*	
*MTHFR* C677T	CC	CT	TT	CC	CT	TT	C	T	C	T	-	*-*	-
Thawnashom *et al.* (2005) [4]	67	-	23 ^e^	34	-	16 ^e^	NA	NA	NA	NA	NA	NA	90/50
Terruzzi *et al.* (2007) [5]	18	54	12	14	33	5	90	78	61	43	4.9466	0.026	84/52
Lewis *et al.* (2008) [6] ^a^	360	410	112	1165	1086	283	1130	634	3416	1652	1.5459	0.214	882/2534
Lewis *et al.* (2008) [6] ^b^	163	155	38	2707	2713	715	481	231	8127	4143	0.7882	0.375	356/6135
Lewis *et al.* (2008) [6] ^c^	115	93	25	2155	2190	552	323	143	6500	3294	0.0153	0.902	233/4897
Lewis *et al.* (2008) [6] ^d^	588	574	107	3812	3356	736	1750	788	10,980	4828	0.0045	0.946	1269/7904
Settin *et al.* (2009) [7]	89	34	5	69	36	5	212	44	174	46	0.0121	0.912	128/110
Bazzaz *et al.* (2010) [8]	44	21	9	113	80	14	109	39	306	108	0.0009	0.975	74/207
Gara *et al.* (2011) [9]	15	14	2	9	12	1	44	18	30	14	1.4550	0.228	31/22
Chauhan *et al.* (2012) [10]	348	185	29	272	148	16	881	243	692	180	0.5680	0.451	562/436
Yin *et al.* (2012) [11]	354	341	56	471	441	66	1049	453	1383	573	7.6608	0.006	751/978
Tabassum *et al.* (2012) [12]	290	144	20	581	218	31	724	184	1380	280	0.8313	0.068	454/830
Our study	115	244	158	160	375	206	474	560	695	787	0.1911	0.662	517/741
*MTRR* A66G	AA	AG	GG	AA	AG	GG	A	G	A	G	-	-	-
Terruzzi *et al.* (2007) [5]	39	42	0	36	16	1	120	42	88	18	0.2649	0.607	81/53
Tabassum *et al.* (2012) [12]	110	231	106	244	407	169	451	443	895	745	0.0009	0.976	447/820
Our study	298	186	33	437	251	53	782	252	1125	357	4.0366	0.045	517/741

Abbreviations: *MTHFR*, methylenetetrahydrofolate reductase; *MTRR*, methionine synthase reductase; PCR-RFLP, polymerase chain reaction-restriction fragment length polymorphism; NA, not available; HWE, Hardy–Weinberg equilibrium test; ^a^ British Women’s Heart and Health Study (BWHHS) cohort study; ^b^ Avon Longitudinal Study of Parents and Children (ALSPAC) women cohort study; ^c^ ALSPAC children cohort study; ^d^ Copenhagen City Heart Study (CCHS) cohort study; ^e^ Genotype counts for TT + CT.

**Table 4:** Stratified analysis of the associations of *MTHFR* C677T and *MTRR* A66G polymorphisms with being overweight/obesity.

Subgroup	Recessive		Dominant		Homozygous Codominant		Heterozygous Codominant		Allelic Model	
	OR (95% CI)	*P*_h_	OR (95% CI)	*P*_h_	OR (95% CI)	*P*_h_	OR (95% CI)	*P*_h_	OR (95% CI)	*P*_h_
*MTHFR* C677T polymorphism										
Overall	1.05 (0.94–1.16)	0.730	1.05 (0.99–1.13)	0.140	1.06 (0.94–1.18)	0.664	1.06 (0.99–1.13)	0.095	1.05 (1.00–1.10)	0.284
Ethnicity										
Asian	1.18 (0.99–1.41)	0.862	1.04 (0.93–1.16)	0.304	1.16 (0.95–1.42)	0.880	1.02 (0.91–1.15)	0.155	1.07 (0.98–1.16)	0.513
Caucasian	0.99 (0.86–1.13)	0.477	1.04 (0.90–1.20)	0.049	1.02 (0.89–1.18)	0.209	1.05 (0.91–1.21)	0.080	1.02 (0.92-1.13)	0.077
African	1.45 (0.12–17.04)	-	0.74 (0.25–2.23)	-	1.20 (0.10–15.20)	-	0.70 (0.23–2.17)	-	0.88 (0.38–2.03)	-
Source of control										
Population based	1.02 (0.90–1.45)	0.581	1.07 (1.00–1.18)	0.057	1.05 (0.93–1.20)	0.356	1.07 (0.96–1.19)	0.076	1.05 (0.97–1.13)	0.088
Hospital based	1.19 (0.95–1.49)	0.786	0.90 (0.74–1.10)	0.810	1.13 (0.85–1.50)	0.782	0.85 (0.68–1.06)	0.722	1.02 (0.89–1.17)	0.736
Genotyping method										
PCR-RFLP	0.98 (0.82–1.17)	0.261	1.01 (0.85–1.20)	0.596	1.02 (0.85–1.23)	0.434	1.07 (0.97–1.19)	0.397	1.03 (0.96–1.11)	0.911
TaqMan	1.07 (0.93–1.24)	0.729	0.96 (0.80–1.16)	0.026	1.07 (0.91–1.25)	0.356	0.95 (0.79–1.15)	0.040	1.00 (0.88–1.13)	0.054
Others	1.30 (0.86–1.97)	0.909	1.10 (1.00–1.23)	0.377	1.35 (0.88–2.05)	0.974	1.13 (0.94–1.36)	0.191	1.15 (0.99–1.33)	0.450
Sample size										
Large study	1.04 (0.93–1.16)	0.615	1.06 (0.96–1.16)	0.075	1.06 (0.94–1.19)	0.468	1.05 (0.95–1.16)	0.077	1.05 (1.00–1.10)	0.138
Small study	1.48 (0.82–2.67)	0.788	0.83 (0.61–1.12)	0.782	1.39 (0.75–2.59)	0.762	0.78 (0.55–1.11)	0.642	0.98 (0.76–1.25)	0.616
*MTRR* A66G polymorphism										
Overall	1.09 (0.86–1.38)	0.476	1.27 (0.95–1.70)	0.106	1.19 (0.91–1.55)	0.246	1.28 (0.96–1.72)	0.116	1.14 (0.95–1.35)	0.194

Abbreviations: *MTHFR*, methylenetetrahydrofolate reductase; *MTRR*, methionine synthase reductase; OR, odds ratio; *P*_h_, *p*-value for heterogeneity test.

Only three studies were included in the meta-analysis of the *MTRR* A66G polymorphism. The pooled results showed that the *MTRR* A66G polymorphism was not significantly associated with the risk of being overweight/obesity in all of the tested genetic models (recessive model: OR = 1.09, 95% CI = 0.86–1.38, *p* = 0.476; dominant model: OR = 1.27, 95% CI = 0.95–1.70, *p* = 0.106; homozygous codominant model: OR = 1.19, 95% CI = 0.91–1.55, *p* = 0.202; heterozygous codominant model: OR = 1.28, 95% CI = 0.96–1.72, *p* = 0.098; allelic model: OR = 1.14, 95% CI = 0.95–1.35, *p* = 0.055) (Table 4, Figures S7–S11). Moderate heterogeneity was observed in the dominant and heterozygous codominant models (Figures S7–S11). Due to the limited number of studies, meta-regression analysis and subgroup analysis were not performed to explore the sources of heterogeneity. Sensitivity analyses showed that the overall associations between the *MTRR* A66G polymorphism and being overweight/obesity were changed in the dominant, heterozygous codominant and allelic models after excluding our case-control study; therefore, the results should be interpreted cautiously. Publication bias was also not performed due to the limited number of studies.

## 3. Discussion

Homocysteine plays a pivotal role in cell metabolism by virtue of its involvement in the transfer of methyl groups in the activated methyl cycle. This cycle is in charge of global and gene promoter-specific DNA methylation, which is one of several epigenetic mechanisms involved in the regulation of gene expression [5,6,21,22,24,25]. Therefore, the homocysteine metabolism pathway seems to be a promising candidate pathway for obesity. In the current study, we explored the possible relationships of the two common polymorphisms (*MTHFR* C677T and *MTRR* A66G) in homocysteine metabolism genes with overweight/obesity susceptibility.

Initially, we carried out a case-control study in the Han population originating from Tianjin municipality in northern China. To the best of our knowledge, this is the first study exploring the relationships of the two polymorphisms with being overweight/obesity in a Chinese Han population. We observed that the *MTHFR* C677T polymorphism was not significantly associated with being overweight/obesity, which corroborates earlier observations in Danish [6], Tunisian [9], Iranian [8], Saudi [7] and Italian [5] populations. However, a study conducted among a Thai population [4] showed that the *MTHFR* 677T allele carriers had an increased risk of being overweight/obesity. Furthermore, Tabassum and coworkers reported that the 677T allele was associated with a 1.24-fold increased risk of obesity in Indian children [12], and Lambrinoudaki and colleagues observed that Greek women carrying the 677CT or 677TT genotype had higher BMI and waist hip ratio compared with women carrying the 677CC genotype [29]. We also did not observe any significant association between the *MTRR* A66G polymorphism and overweight/obesity susceptibility. In contrast to our observation, Terruzzi and colleagues reported that Italian adults carrying the 66AG genotype had a 2.42-fold higher risk of obesity than 66AA carriers [5]. Another study of an Indian children sample found a significant association between the 66G allele and obesity [12].

Meta-analysis is the most commonly used statistical technique in medical research, which combines several individual studies in an effort to achieve higher statistical power, to improve the precision of estimates and to settle uncertainty from conflicting individual results [30]. Given the inconsistent results of the studies mentioned above, we performed a comprehensive meta-analysis combining our case-control study and previously published studies to provide more empirical evidence on the associations of the *MTHFR* C677T and *MTRR* A66G polymorphisms with being overweight/obesity. For the *MTHFR* C677T polymorphism, thirteen studies with 5431 cases and 24,896 controls were finally included in the meta-analysis. The overall pooled results showed no significant association of the *MTHFR* C677T polymorphism with overweight/obesity susceptibility and with minimal observed heterogeneity, which confirmed our case-control observations in the Chinese Han population. Subsequent stratified analyses based on ethnicity, source of controls, genotyping method and sample size also did not observe any significant association in all of the subgroups. Sensitivity analyses revealed that these pooled results were reliable, and a publication bias test showed little evidence of publication bias. All of these results indicated that the *MTHFR* C677T polymorphism might not be a genetic risk factor for being overweight/obesity. For the *MTRR* A66G polymorphism, only three studies with 1045 cases and 1614 controls were included in the meta-analysis. The overall pooled results also showed no significant association of the *MTRR* A66G polymorphism with being overweight/obesity. Due to the limited number of studies, we did not perform subgroup analysis, meta-regression analysis and publication bias assessment. However, we performed sensitivity analyses and found that the association between the *MTRR* A66G polymorphism and being overweight/obesity became significant in three genetic models after excluding our present case-control study. This indicates that our case-control study had a great impact on the overall estimates. It must be noted that the *MTRR* A66G polymorphism was not in HWE in the controls, which could have affected the results of our case-control study. Thus, a representative sample is still needed to verify a possible association of this polymorphism with being overweight/obesity in the Chinese population, and the meta-analytic results for the *MTRR* A66G polymorphism should be interpreted with great caution.

In interpreting the findings of the current study, several limitations should be acknowledged. Firstly, being overweight/obesity was defined by BMI in our case-control study, which is limited by not being able to distinguish between fat mass and lean or bone mass, and it does not reflect fat distribution. In addition, lack of information on other anthropometric measurements and direct measurements of body composition precluded us from providing a more comprehensive estimation of the two polymorphisms with body composition; especially, several studies have suggested a link between *MTHFR* gene polymorphism and reduced muscle mass [31,32]. Secondly, the meta-analysis concerning the *MTHFR* C677T polymorphism had sufficient statistical power and confirmed the results of our case-control study; however, the meta-analysis on the *MTRR* A66G polymorphism is underpowered, because of the small sample size. Thirdly, the subjects of our case-control study were from one hospital, which might not possess adequate representation. Fourthly, the included articles were limited to those published in Chinese and English, thus publication bias might have occurred, although funnel plots and Begg’s test showed no evidence of publication bias. Despite these limitations, our study still had its own special advantages. Firstly, it explored the associations of the *MTHFR* C677T and *MTRR* A66G polymorphisms with being overweight/obesity for the first time in the Chinese Han population. Secondly, it adopted a comprehensive analysis strategy by integrating case-control study and meta-analysis together, thus enlarging the sample size and strongly enhancing the study power. Thirdly, the heterogeneity test, publication bias assessment and sensitivity analyses indicated that our results for the *MTHFR* C677T polymorphism were statistically robust and reliable.

## 4. Experimental Section

### 4.1. Study Subjects

Between October 2008 and February 2011, a total of 517 overweight/obesity subjects and 741 normal weight subjects who took regular health examinations at the physical examination center of Dagang Oil Field General Hospital were recruited into our study. Participants with non-communicable disease history, endocrine diseases and any other diseases that might influence our results were excluded. According to the Guidelines on the Prevention and Management of Overweight and Obesity in Adults: China, normal weight was defined as a BMI <24.0, overweight was defined as 24 ≤ BMI < 28, and obesity was defined as a BMI ≥28. All patients and controls were unrelated and of the same ethnic background. The study was conducted in accordance with the principles stipulated by the Declaration of Helsinki and were approved by the ethics review committee of China Medical University (Shenyang, China; Identification Code: CMU62073024; 15 July 2008).

### 4.2. Clinical Measurements and Laboratory Tests

Body weight, height and WC were measured using a standard scale with light clothing and barefoot after an overnight fast. BMI was calculated as weight in kilograms divided by the square of height in meters (kg/m^2^). Blood pressure was measured while subjects were in the sitting position after 15 min of rest. The average of three measurements was recorded. At the same time, participants were asked for permission to store a blood sample for biochemical analysis and a buccal cell sample for genetic analysis. The levels of TC, HDL-C, LDL-C, TG and FBG in samples were measured using a Hitachi autoanalyzer (Type 7170A; Hitachi Ltd., Tokyo, Japan).

### 4.3. Genotyping Analysis

Genomic DNA was extracted from buccal samples using the QIAamp DNA Mini Kit (Qiagen, Valencia, CA, USA). Genotyping for the *MTHFR* C677T and *MTRR* A66G polymorphisms was performed as described previously [28].

### 4.4. Statistical Analysis

The *MTHFR* C677T and *MTRR* A66G allele and genotype frequencies in the cases and controls were calculated by direct counting. Chi-square analysis was performed to identify departures from HWE and to compare the difference between the two groups with respect to allelic and genotypic frequencies. The unconditional logistic regression analysis was performed to estimate the effects of the two polymorphisms on overweight/obesity risk after adjustment for age and gender. OR with 95% CI was calculated to estimate the relative risk of the different genotypes and alleles. A *p*-value below 0.05 was taken as statistically significant. These analyses were performed using SAS Version 9.2 (SAS Institute, Cary, CN, USA).

### 4.5. Meta-Analysis

We systematically searched three English (PubMed, Web of Science and Embase) and four Chinese databases (China National Knowledge Infrastructure (CNKI), Wanfang, China Biological Medicine Database (CBM) and Chongqing VIP Chinese Science and Technology Periodical Database (VIP)) for studies exploring the associations of the *MTHFR* C677T and/or *MTRR* A66G polymorphisms with being overweight and/or obesity. Search strategies were based on combinations of the following key words: obesity, adiposity, obese, overweight, *MTHFR*, methylenetetrahydrofolate reductase, *MTRR*, methionine synthase reductase, allele, gene, genotype, variant, variation and polymorphism. The reference lists of retrieved articles were hand-searched to find potentially eligible studies. All of the included studies had to meet the following criteria: (1) evaluating the associations of the *MTHFR* C677T and/or *MTRR* A66G polymorphisms with being overweight and/or obesity; (2) case-control or cohort studies in design; and (3) OR with 95% CI could be obtained or calculated with genotype data.

Two curators independently extracted the following information from each included study: first author’s name, year of publication, ethnicity and country of study population, source of controls, genotyping method, diagnostic criteria of overweight and obesity, mean age, gender proportion, counts of alleles and genotypes and the numbers of cases and controls. If there was any discrepancy, an agreement was reached by discussion between the investigators.

The HWE in the control groups was tested again. The associations of the *MTHFR* C677T and *MTRR* A66G polymorphisms with being overweight/obesity were assessed by calculating pooled ORs with their corresponding 95% CIs under the homozygous codominant, heterozygous codominant, dominant, recessive and allelic models [27]. The significance of the pooled OR was determined by the Z test. Between-study heterogeneity was assessed by Cochran’s chi-square based Q-test and the *I*^2^ statistic [33,34]. *p* < 0.05 for the Q-test or *I*^2^ > 50% was considered with significant heterogeneity. Once the effects were assumed to be heterogeneous, the random effects model was used; otherwise, the fixed effects model was applied [35]. Subgroup analyses by ethnicity, source of control, genotyping method and sample size were also performed. Sensitivity analysis was conducted to assess the stability of the results [36]. Publication bias was assessed using the funnel plot and Egger’s regression test [37]. All statistical analysis was performed using the STATA package Version 11.0 program (StataCorp, College Station, TX, USA), and a *p*-value less than 0.05 was considered to be statistically significant.

## 5. Conclusions

In conclusion, our case-control study in combination with the following meta-analysis suggests that neither the *MTHFR* C677T nor *MTRR* A66G polymorphism is associated with overweight/obesity risk. However, considering the limitations mentioned before, further large-scale population-based studies, especially for the *MTRR* A66G polymorphism, are still needed to confirm or refute our findings. We hope that these results will provide background data for the future study of obesity pathogenesis and will contribute to genetic marker screening.

## Supplementary Materials

Supplementary materials can be found at http://www.mdpi.com/1422-0067/16/06/11849/s1.
