# Supplementary material for: Are MTHFR C677T and MTRR A66G Polymorphisms Associated with Overweight/Obesity Risk? From a Case-Control to a Meta-Analysis of 30,327 Subjects
Source: Int J Mol Sci. 2015 May 26;16(6):11849–63. doi: 10.3390/ijms160611849 (PMC4490418; doi:10.3390/ijms160611849)
Supplement: Supplementary file 1 [file ijms-16-11849-s001.pdf]

Supplementary Information

Table S1. Baseline characteristics of the studies included in the meta-analysis.

Author (Year) [Ref.]	Country (Ethnicity)	Genotyping Method	Mean Age (Cases/Controls)	Source of Controls	Diagnose Criteria	BMI (Cases; Controls)	SNPs Studied
Thawnashom *et al.* (2005) [4]	Thailand (Asian)	PCR-RFLP	40/38	HB	BMI ≥ 25	31.5 (25.1–56.2); 21.8 (18.52–24.97) *	*MTHFR* C677T
Terruzzi *et al.* (2007) [5]	Italy (Caucasian)	PCR-RFLP	43.3/35.2	HB	BMI ≥ 30	34.1 ± 7.1; 22.4 ± 1.8 [#]	*MTHFR* C677T; *MTRR* A66G
Lewis *et al.* (2008) [6] [a]	English (Caucasian)	TaqMan	68.8 [e]	PB	BMI ≥ 30	None	*MTHFR* C677T
Lewis *et al.* (2008) [6] [b]	English (Caucasian)	TaqMan	28.5 [e] (Young women)	PB	BMI ≥ 30	None	*MTHFR* C677T
Lewis *et al.* (2008) [6] [c]	English (Caucasian)	TaqMan	9.9 [e] (Children)	PB	Females: BMI > 23.46 Males: BMI > 23.39	None	*MTHFR* C677T
Lewis *et al.* (2008) [6] [d]	Danish (Caucasian)	PCR-RFLP	57.6 [e]	PB	BMI ≥ 30	None	*MTHFR* C677T
Settin *et al.* (2009) [7]	Saudi Arabia (Asian)	TaqMan	27/24	HB	BMI ≥ 25	27 ± 9.8; 24 ± 8.8 [#]	*MTHFR* C677T
Bazzaz *et al.* (2010) [8]	Iran (Asian)	PCR-RFLP	44.8/41.6	HB	BMI ≥ 30	33.8 ± 3.4; 24.8 ± 2.9 [#]	*MTHFR* C677T
Gara *et al.* (2011) [9]	Tunisia (African)	Pyrosequencing	4.6–14.3/NA	HB	BMI > 97% percentile	None	*MTHFR* C677T
Chauhan *et al.* (2012) [10]	India (Asian)	GoldenGate assay	44–64 [e]	PB	BMI ≥ 23	None	*MTHFR* C677T
Yin *et al.* (2012) [11]	China (Asian)	PCR-RFLP	41.25/41.48	PB	BMI ≥ 24	26.48 ± 2.59; 21.23 ± 1.68 [#]	*MTHFR* C677T
Tabassum *et al.* (2012) [12]	India (Asian)	GoldenGate assay	13.00/14.00	PB	BMI ≥ 25	25.85 (23.97–28.97); 17.58 (15.86–19.44) *	*MTHFR* C677T, *MTRR* A66G

Abbreviation: *MTHFR*, methylenetetrahydrofolate reductase; *MTRR*, methionine synthase reductase; PCR-RFLP, polymerase chain reaction-restriction fragment length polymorphism; HB, hospital based; PB, population based; BMI, body mass index; NA, not available; [a] BWHHS cohort study; [b] ALSPAC women cohort study; [c] ALSPAC children cohort study; [d] CCHS cohort study; [e] The mean age of all subjects; * Data are presented as the median (25th–75th); [#] Data are presented as the mean ± SD.

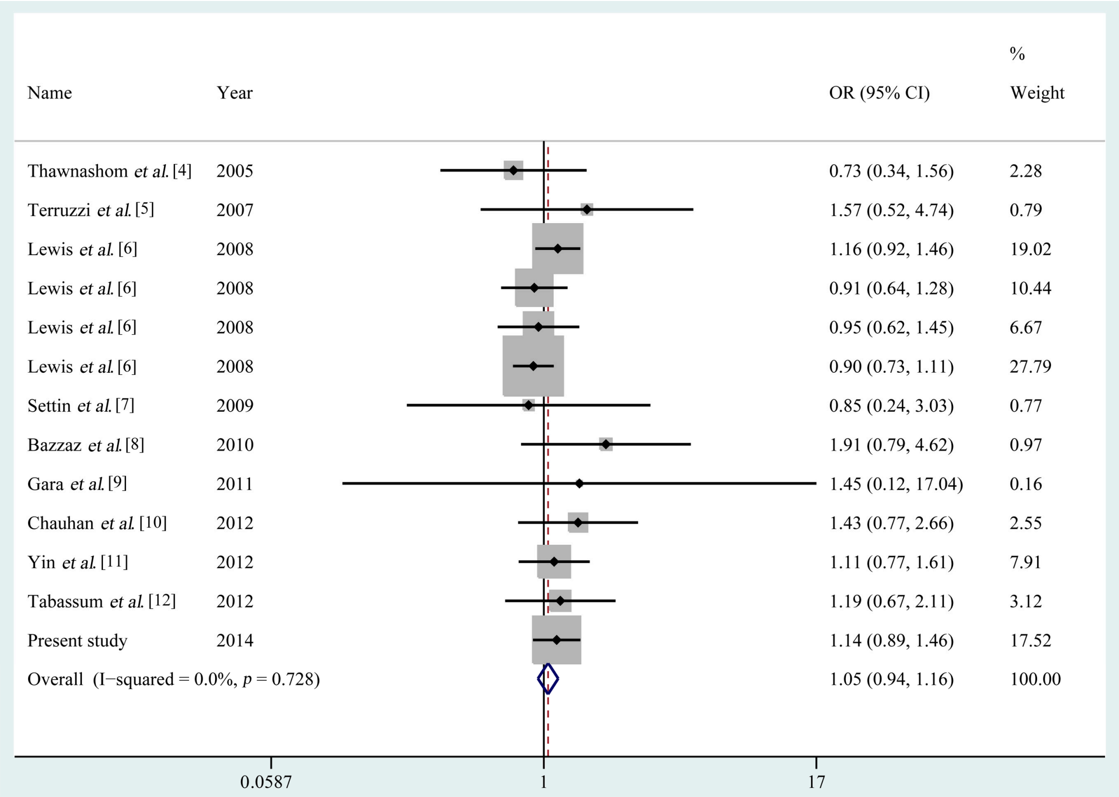

Figure S1. Forest plot of the association between the *MTHFR* C677T polymorphism and being overweight/obesity in the recessive model (TT *vs.* CT + CC).

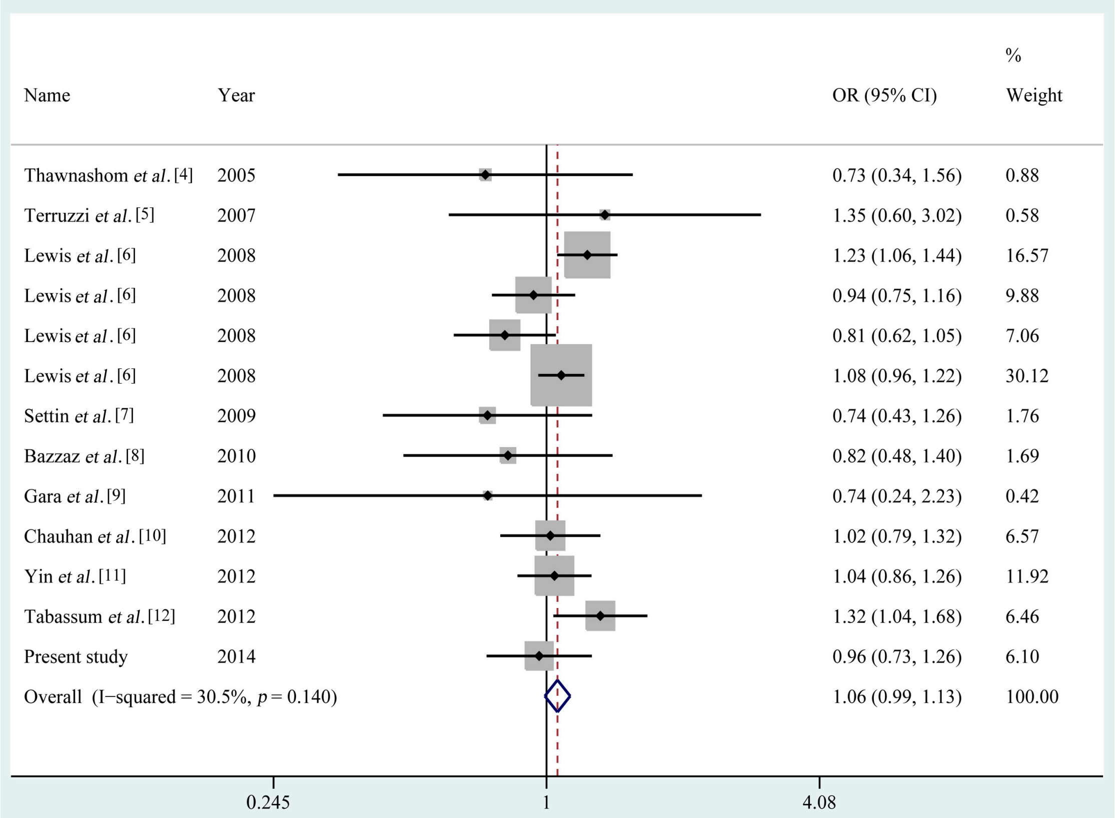

Figure S2. Forest plot of the association between the *MTHFR* C677T polymorphism and being overweight/obesity in the dominant model (TT + CT *vs.* CC).

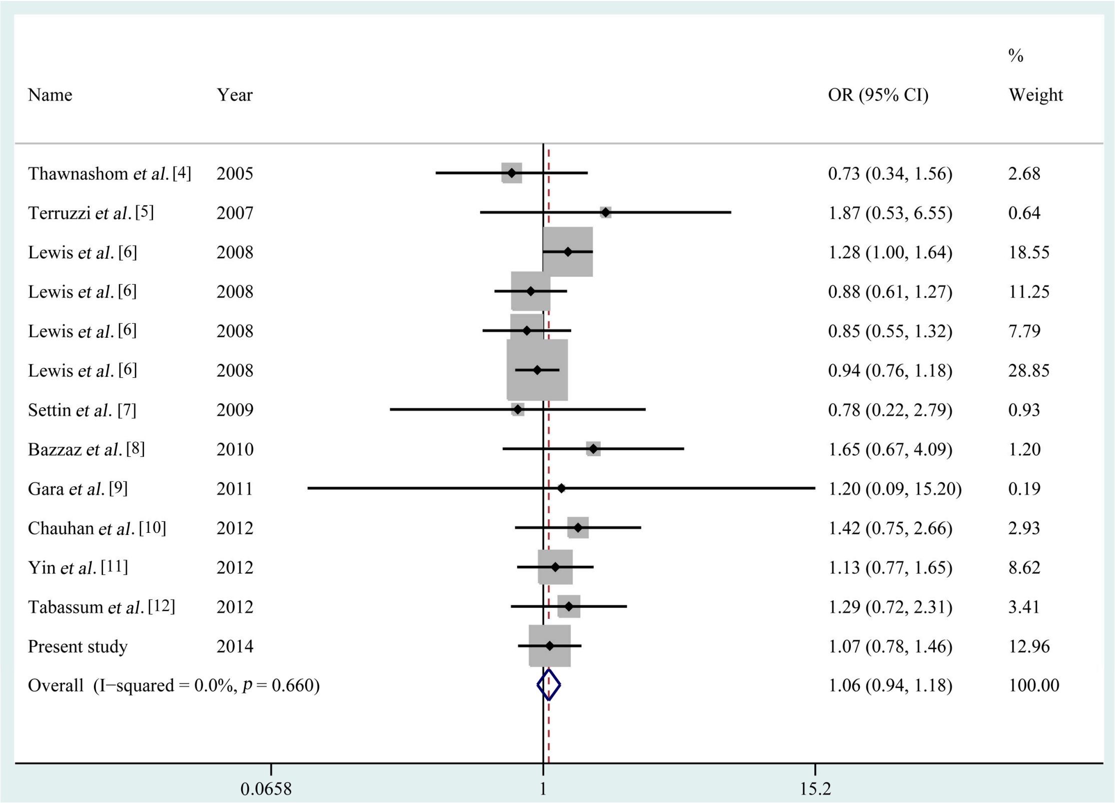

Figure S3. Forest plot of the association between the *MTHFR* C677T polymorphism and being overweight/obesity in the homozygous codominant model (TT *vs*. CC).

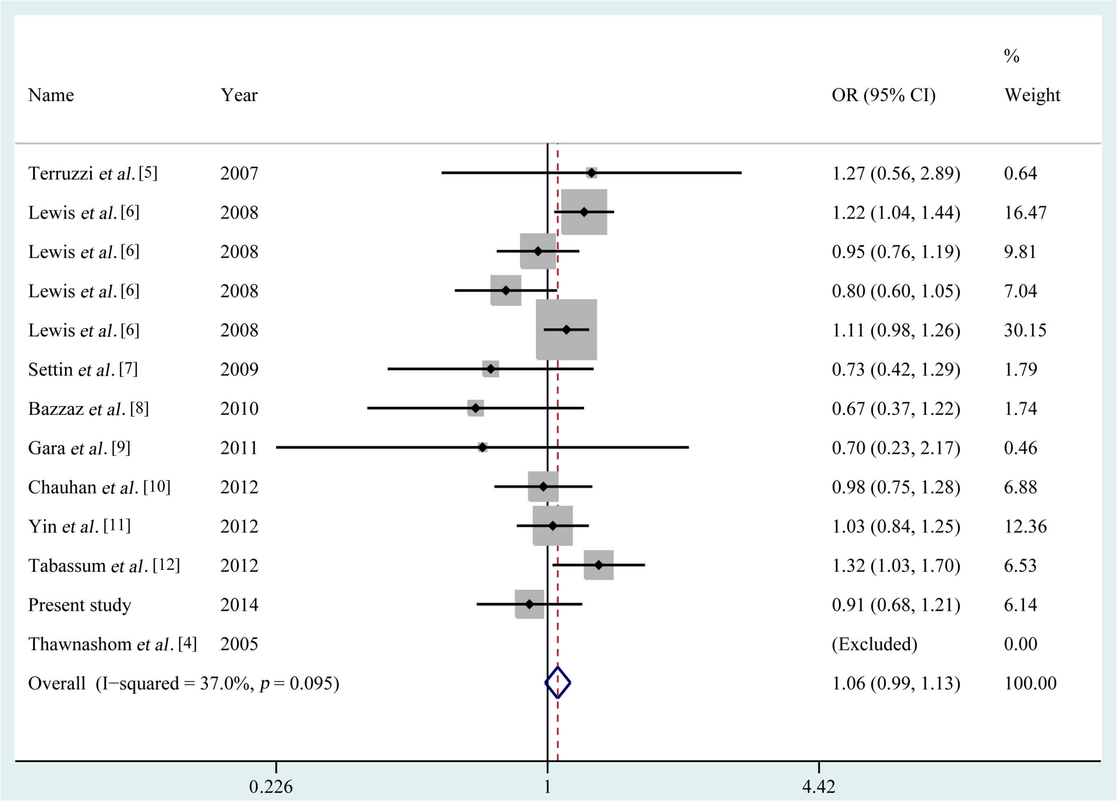

Figure S4. Forest plot of the association between the *MTHFR* C677T polymorphism and being overweight/obesity in the heterozygous codominant model (CT *vs*. CC).

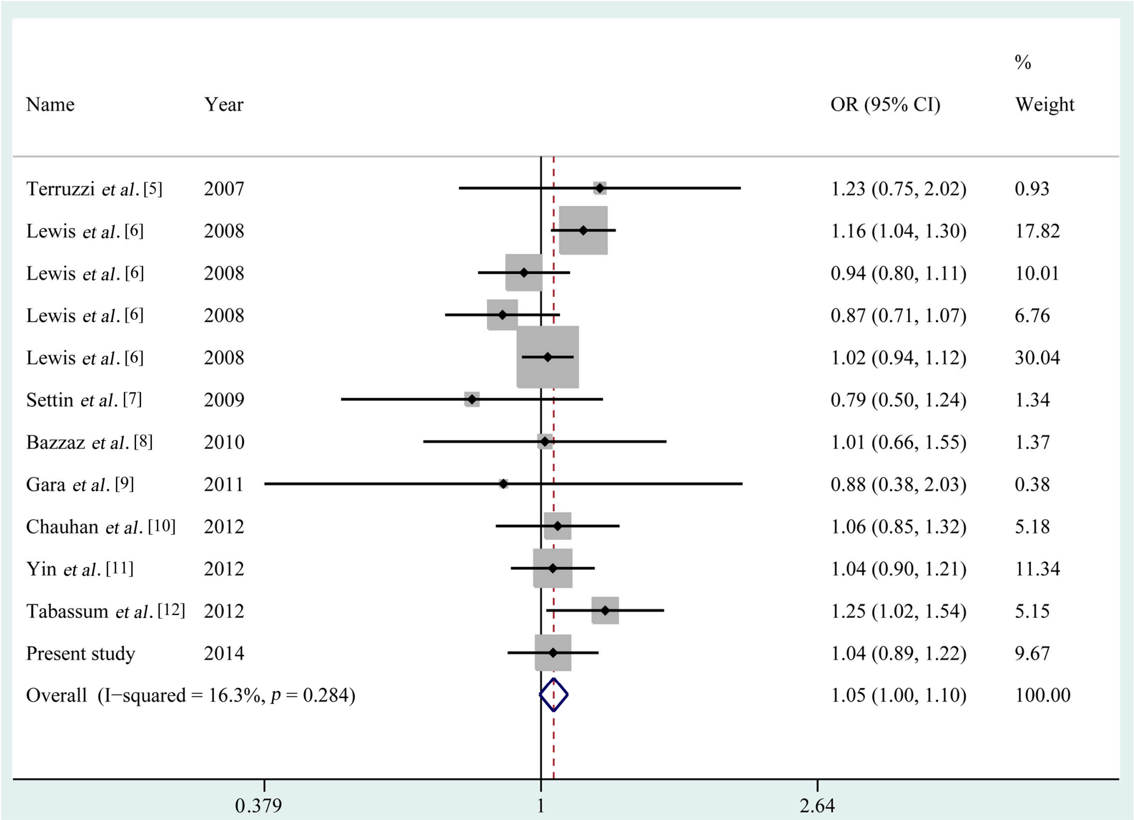

Figure S5. Forest plot of the association between the *MTHFR* C677T polymorphism and being overweight/obesity in the allelic model (T *vs.* C).

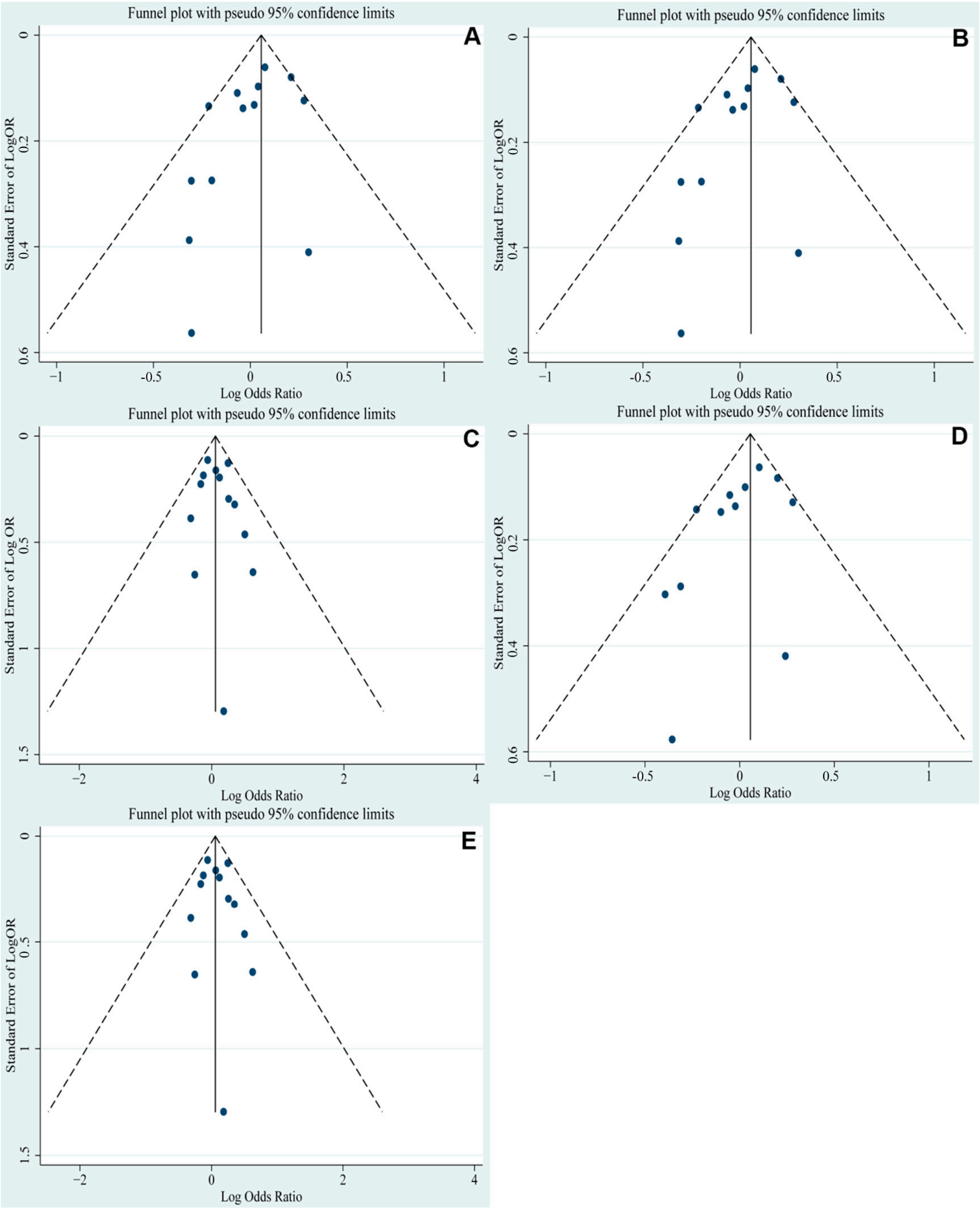

Figure S6. Funnel plot analysis on the detection of publication bias in the meta-analysis of the associations between MTHFR polymorphisms and being overweight/obesity: (**A**) recessive model; (**B**) dominant model; (**C**) homozygous codominant model; (**D**) heterozygous codominant model; (**E**) allelic model.

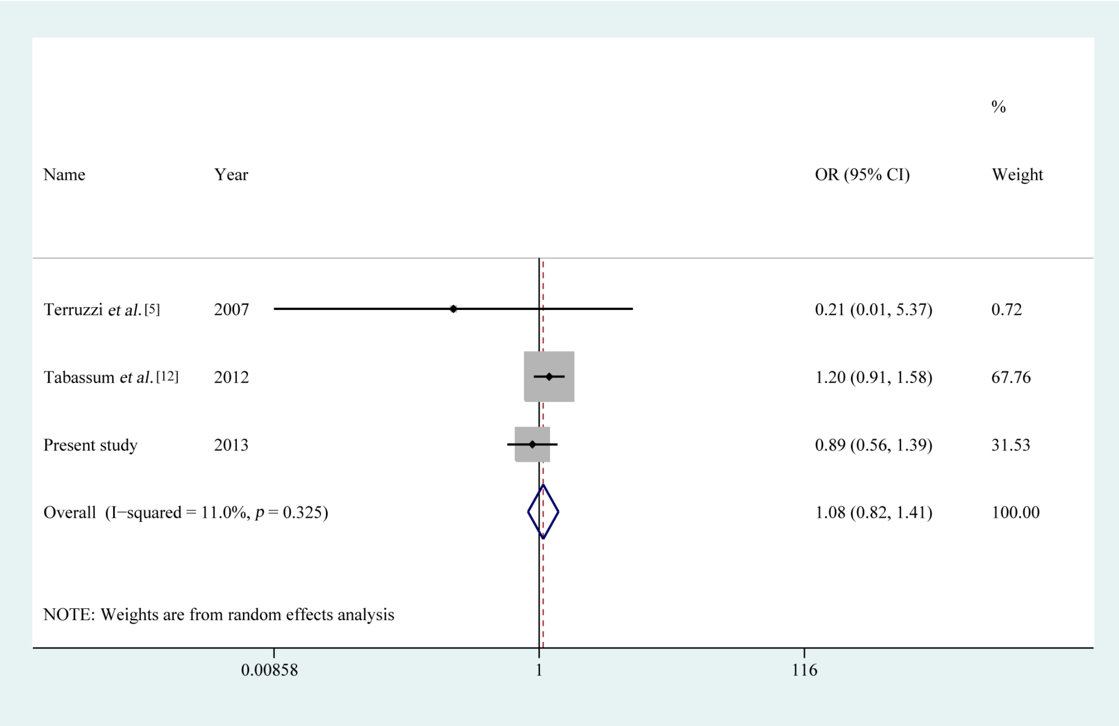

Figure S7. Forest plot of the association between the *MTRR* A66G polymorphism and being overweight/obesity in the recessive model (TT *vs.* CT + CC).

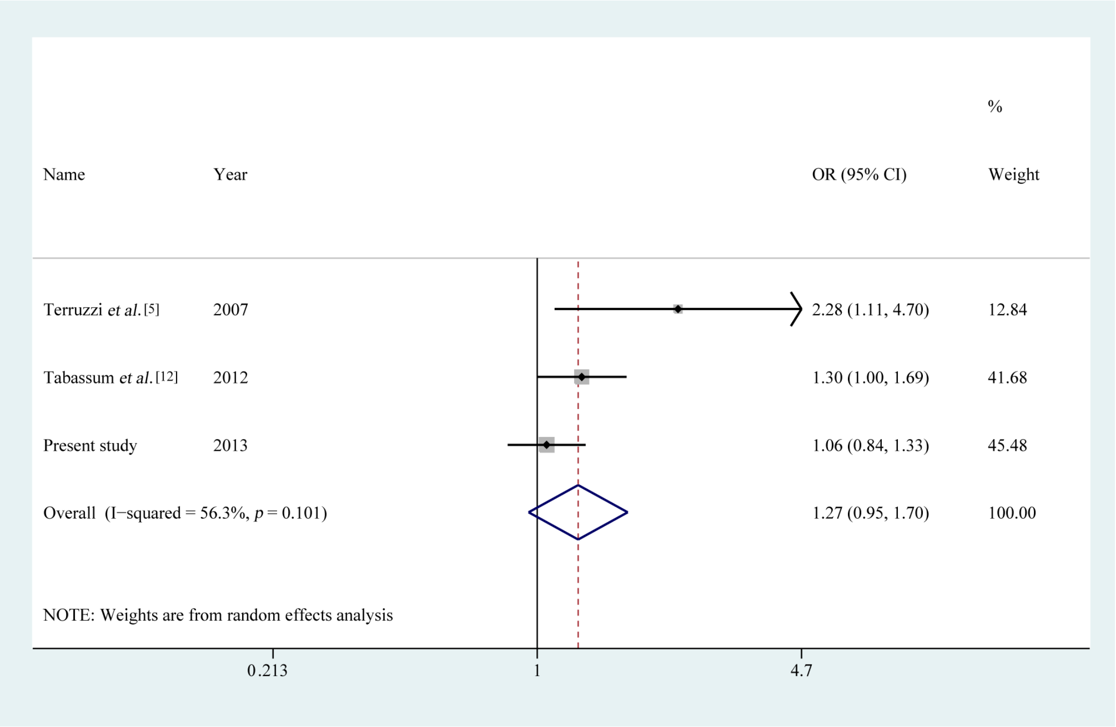

Figure S8. Forest plot of the association between the *MTRR* A66G polymorphism and being overweight/obesity in the dominant model (TT + CT *vs.* CC).

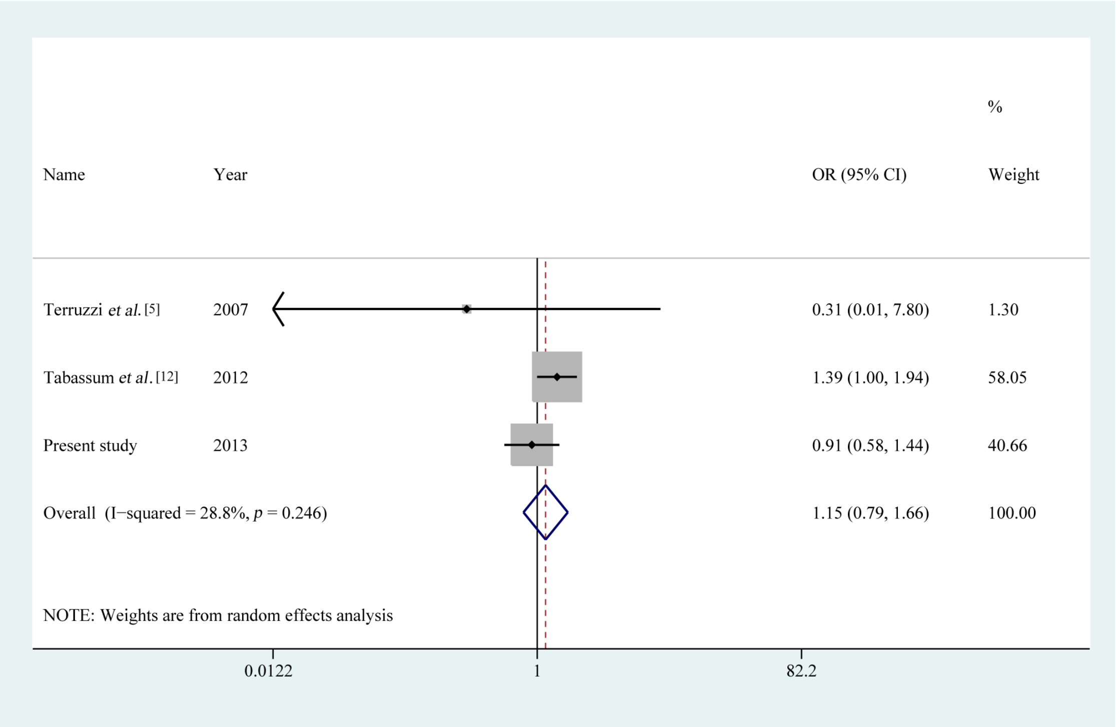

Figure S9. Forest plot of the association between the *MTRR* A66G polymorphism and being overweight/obesity in the homozygous codominant model (TT *vs.* CC).

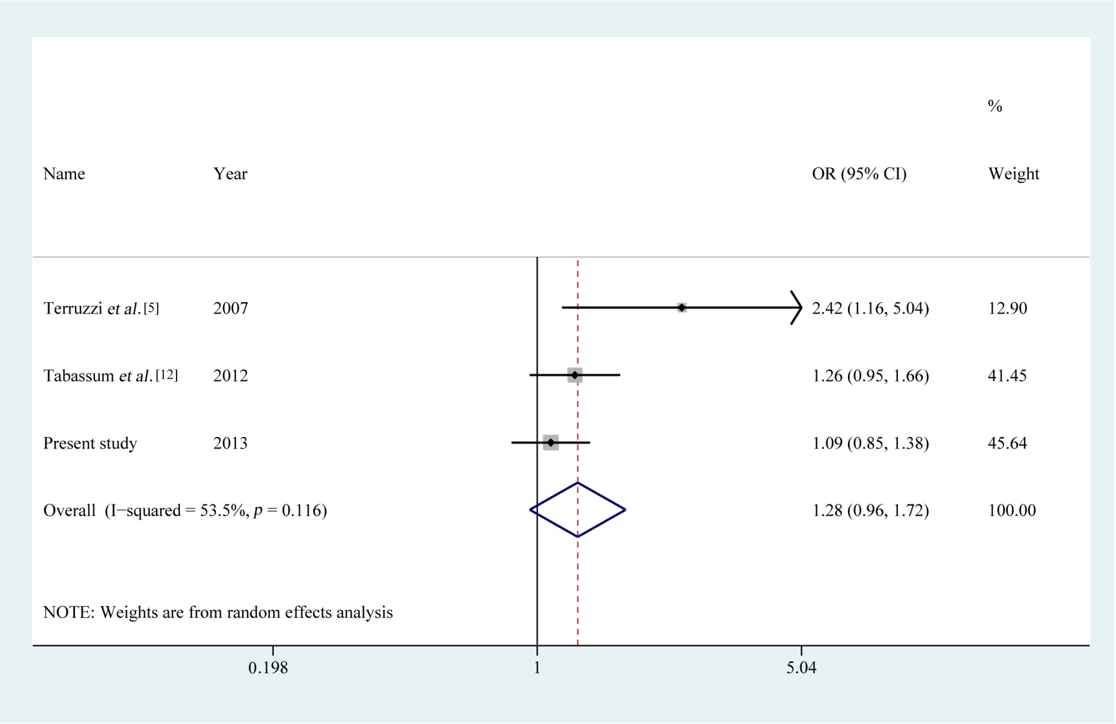

Figure S10. Forest plot of the association between the *MTRR* A66G polymorphism and being overweight/obesity in the heterozygous codominant model (CT *vs.* CC).

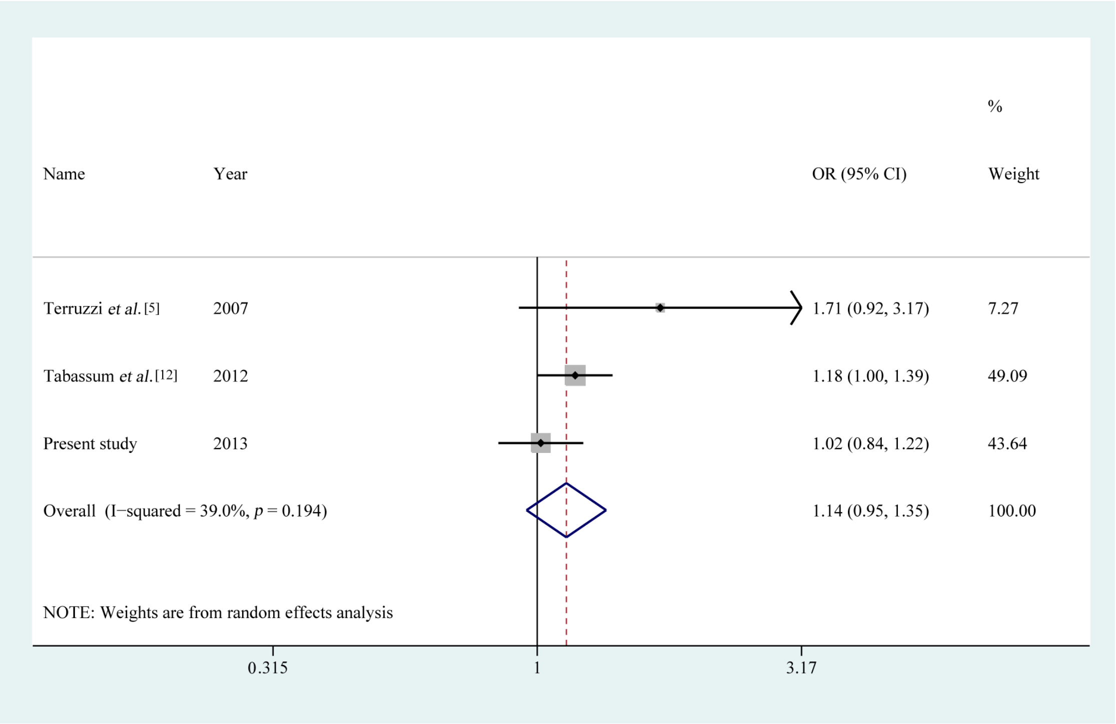

Figure S11. Forest plot of the association between the *MTRR* A66G polymorphism and being overweight/obesity in the allelic model (T *vs.* C).
